# FOXC1 Binds Enhancers and Promotes Cisplatin Resistance in Bladder Cancer

**DOI:** 10.3390/cancers14071717

**Published:** 2022-03-28

**Authors:** Yi-Tsung Lu, Tong Xu, Maheen Iqbal, Tien-Chan Hsieh, Zhifei Luo, Gangning Liang, Peggy J. Farnham, Suhn K. Rhie, Amir Goldkorn

**Affiliations:** 1 Division of Medical Oncology, Department of Medicine, Norris Comprehensive Cancer Center, Keck School of Medicine, University of Southern California, Los Angeles, CA 90033, USA; yi-tsung.lu@med.usc.edu (Y.-T.L.); tongxu@usc.edu (T.X.); maheeniq@usc.edu (M.I.); tien-chan.hsieh@nuvancehealth.org (T.-C.H.); 2 Department of Medicine, Danbury Hospital, Danbury, CT 06810, USA; 3 Department of Biochemistry and Molecular Medicine, Norris Comprehensive Cancer Center, Keck School of Medicine, University of Southern California, Los Angeles, CA 90033, USA; zhl116@eng.ucsd.edu (Z.L.); peggy.farnham@med.usc.edu (P.J.F.); 4 Department of Bioengineering, University of California, San Diego, CA 92093, USA; 5 Department of Urology, Keck School of Medicine, University of Southern California, Los Angeles, CA 90033, USA; gliang@usc.edu

**Keywords:** FOXC1, chromatin accessibility, drug resistance, enhancer activation, bladder cancer

## Abstract

**Simple Summary:**

In bladder cancer, cisplatin remains the front-line therapy, but drug resistance is common. Previously, we showed that cancer cells can spontaneously convert to an aggressive drug-resistant phenotype without mutational events. In the current work, we explored the epigenetic mechanism behind the conversion to the drug-resistant phenotype. We discovered that drug-resistant cells have differentially accessible enhancers, which are bound by FOXC1, a transcription factor that is overexpressed in these cells. Accordingly, FOXC1 knockout significantly attenuates the emergence of the drug-resistant phenotype and reduces cell survival upon cisplatin treatment. These findings suggest that FOXC1 binding at accessible enhancers promotes cisplatin drug resistance in bladder cancer cells. Therefore, FOXC1 targeting may be a new therapeutic avenue to mitigate cisplatin resistance and improve treatment efficacy in bladder cancer.

**Abstract:**

Chemotherapy resistance is traditionally attributed to DNA mutations that confer a survival advantage under drug selection pressure. However, in bladder cancer and other malignancies, we and others have previously reported that cancer cells can convert spontaneously to an aggressive drug-resistant phenotype without prior drug selection or mutational events. In the current work, we explored possible epigenetic mechanisms behind this phenotypic plasticity. Using Hoechst dye exclusion and flow cytometry, we isolated the aggressive drug-resistant cells and analyzed their chromatin accessibility at regulatory elements. Compared to the rest of the cancer cell population, the aggressive drug-resistant cells exhibited enhancer accessibility changes. In particular, we found that differentially accessible enhancers were enriched for the FOXC1 transcription factor motif, and that FOXC1 was the most significantly overexpressed gene in aggressive drug-resistant cells. ChIP-seq analysis revealed that differentially accessible enhancers in aggressive drug-resistant cells had a higher FOXC1 binding, which regulated the expression of adjacent cancer-relevant genes like *ABCB1* and *ID3*. Accordingly, cisplatin treatment of bladder cancer cells led to an increased FOXC1 expression, which mediated cell survival and conversion to a drug-resistant phenotype. Collectively, these findings suggest that FOXC1 contributes to phenotypic plasticity by binding enhancers and promoting a mutation-independent shift towards cisplatin resistance in bladder cancer.

## 1. Introduction

Bladder cancer is the eighth leading cause of cancer death in men, with an estimated 17,100 deaths in both men and women in the US in 2022 [1]. While it remains one of the most lethal cancers, significant advances in the understanding of the disease have led to the introduction of novel therapies altering the treatment landscape [2]. Despite the development of novel therapeutics and a better understanding of the disease process [3], cisplatin-based chemotherapy remains the backbone of standard systemic therapy. However, while some patients have an initial response to treatment, most ultimately relapse and succumb to their disease. The emergence of cisplatin resistance, therefore, remains a major obstacle to lasting cures.

While DNA mutations have long been the focus of resistance studies, there is now accumulating evidence that non-mutational mechanisms also play a role in cancer drug resistance. We have been studying a subpopulation of highly drug-resistant, tumorigenic side population (SP) cells in bladder cancer that emerge cyclically and predictably from non-side population (NSP) cells through serial passages in culture [4,5,6]. We have demonstrated that conversion to this drug-resistant SP phenotype is mediated, at least in part, by PIK3CA/AKT signaling and CBP/β-catenin transcriptional activation without additional mutations [5]. Similar non-mutational mechanisms of therapeutic resistance have been observed in other cancer types. In melanoma, a drug resistance subpopulation was driven by an epigenetic program induced upon drug treatment [7]. In colon cancer, cancer stem-like cells spontaneously emerged from the non-stem cells and were enriched after chemotherapy treatment [8]. More recently, single-cell sequencing identified a group of drug-resistant cancer cells that were able to undergo transcriptional adaptation and transcriptome reprogramming under selection pressure from chemotherapy treatment [9]. Collectively, these observations established that non-mutational mechanisms contribute to the emergence of drug-resistant cancer cells from the drug-susceptible overall population.

It has been postulated that the emergence of drug resistance is mediated by epigenetic mechanisms. Epigenetic perturbations such as enhancer mutation and enhancer hijacking have been demonstrated to play important roles in oncogenic transformation and cancer progression [10]. Furthermore, the expression of some oncogenes can be controlled by modulating chromatin modifiers and subsequent enhancer-promoter interactions. In several studies, chromatin modifiers in the KDM (histone lysine demethylase) family were shown to induce a slow-cycling, drug-resistant phenotype in a broad spectrum of malignancies [7,11,12,13]. In our model, we previously found that E2F3, a transcription factor crucial to the SP phenotype, has a more accessible promoter and is overexpressed in J82 SP cells [6]. Based on these earlier findings, we hypothesized that a genome-wide accessibility change exists when cells transit from NSP to SP cells, and we undertook a comprehensive investigation of differential chromatin accessibility at regulatory elements between SP and NSP cells. Using DNA methylation, chromatin immunoprecipitation coupled with sequencing (ChIP-seq), and transcriptome profiling, we characterized a potential epigenetic driver that promotes plasticity toward the drug-resistant phenotype.

## 2. Materials and Methods

### 2.1. Cell Culture

Human bladder cancer cell lines J82 (KCLB Cat# 30001, RRID: CVCL_0359), T24 (CCLV Cat# CCLV-RIE 0062, RRID: CVCL_0554), TCCSUP (ATCC Cat# HTB-5, RRID: CVCL_1738), and UMUC-3 (ECACC Cat# 96020936, RRID: CVCL_1783) were maintained in RPMI 1640 (T24) and DMEM (remaining cell lines) (Mediatech, Inc., Manassas, VA, USA) supplemented with 10% heat-inactivated fetal bovine serum (Omega, Tarzana, CA, USA), 1% penicillin (100 units/mL, Invitrogen, Waltham, MA, USA), and 1% streptomycin (100 µg/mL, Thermo Fisher Scientific, Waltham, MA, USA) at 37 °C and 5% CO_2_ [4,5]. We authenticated the cell lines using 9-marker STR profiling (IDEXX BioAnalytics, Columbia, MO, USA) within the past 3 years. Interspecies contamination test (IDEXX BioAnalytics , Columbia, MO, USA) and mycoplasma evaluation (MycoAlert mycoplasma detection kit, Lonzo, Basel, Switzerland) were negative.

### 2.2. SP Assays

Hoechst staining and FACS analysis and sorting were conducted as described previously [4,5]. Briefly, cells were trypsinized, counted, and resuspended in prewarmed media at a concentration of 1 × 10^6^/mL. Hoechst 33342 (Sigma-Aldrich, St. Louis, MO, USA) was added at a concentration of 5 μg/mL, incubated for 2 h in a 37 °C water bath, and gently inverted several times during incubation. The cells were washed and resuspended in ice-cold DMEM media. Then, 7-AAD, used to discriminate dead cells, was added to the cells at a final concentration of 2 μg/mL. Samples were incubated for at least 5 min on ice before FACS analysis and sorting (FACSAria and FACSymphony, Tha, San Diego, CA, USA; both equipped with UV lasers).

### 2.3. AcceSssIble Assays

The AcceSssIble assay used in this paper was previously published along with the detailed methods [6]. Briefly, purified nucleic acids from SP and NSP cells were separated into the CpG methyltransferase (M.SssI, New England BioLabs, Ipswich, MA, USA) treatment group and the no enzyme control group. The M.SssI-treated groups gained methylation at accessible sites compared to the control groups [14,15,16]. The subsequent Infinium DNA methylation assay was performed at the University of Southern California Molecular Genomics Core, according to the manufacturer’s specifications (Illumina MethylationEPIC BeadChip, Illumina, San Diego, CA, USA). Beta (β) values from each methylation probe were calculated using the minfi package preprocessNoob function [17]. The accessibility of a probe was defined as the difference between the β-value of M.SssI-treated groups and the β-value of the control group. Following the original publication, a probe is defined as accessible if the increase of β-value after M.SssI-treatment is more than 0.3 [16].

### 2.4. RNA Sequencing (RNA-seq)

After RNA extraction by the Direct-zol RNA Kit (Zymo Research, Irvine, CA, USA), the RNA integrity number was measured by the Agilent 2100 Bio-Analyzer. For the RNA sequencing of SP and NSP cells, libraries were constructed using the KAPA mRNA HyperPrep Kit (Roche, Basel, Switzerland) using NEXTflex DNA Barcodes (Bioo Scientific, Austin, TX, USA). RNA sequencing was performed at the UCLA Technology Center for Genomics and Bioinformatics (Los Angeles, CA, USA), using an Illumina HiSeq 3000 platform (Illumina) for single-end sequencing, 50-bp read length, for about 30 million raw reads per sample.

For the RNA sequencing of J82 FOXC1-knockout and vector control cells, libraries were constructed using the KAPA mRNA HyperPrep Kit (Roche), and the sequencing was performed at the University of Southern California Molecular Genomics Core using NextSeq 550 (Illumina) platform for pair-end sequencing, 75-bp read length, for about 30 million raw reads per sample.

The resulting RNA-seq data were aligned to the hg19 human genome build using the STAR aligner [18]. SAM files were converted to BAM files using Samtools [19]. Counts for each gene from gencode version 19 [20] were assigned using the Subread package’s feature Counts program, with BAM files as the inputs [21]. Read counts were normalized and analyzed for differential expression analysis using the DESeq2 package in R 4.0.2 [22]. The cutoff of significantly differentially expressed genes was an adjusted *p*-value < 0.05 based on the Benjamini−Hochberg Procedure and a fold change of more than 30%. Transcripts with low mean normalized counts were filtered by DESeq2 as per the default setting. Gene set enrichment analysis was implemented and visualized using the clusterProfiler package [23].

### 2.5. Chromatin Immunoprecipitation Coupled with Sequencing (ChIP-seq)

For the J82 and T24 H3K27ac ChIP-seq analyses, raw data were downloaded from Gene Expression Omnibus (GEO), accession number GSE75286 [24]. ChIP-seq data were mapped to hg19 using bwa [25], and the peaks were called using MACS2 [26] according to the ENCODE3 ChIP-seq pipeline (https://www.encodeproject.org/chip-seq/ accessed on 15 May 2019). Enrichment peaks were examined in IGV, and the true H3K27ac peaks in J82 and T24 were visually determined by the enrichment *p* value based on the comparison of the peak to background. We determined the top 60,000 H3K27ac binding sites as true H3K27ac binding sites in J82, and the top 25,000 H3K27ac binding sites in T24. All true peaks, which had enrichment q-values < 1 × 10^−5^, , were used for the downstream analyses (Supplementary Table S1).

FOXC1 Chromatin immunoprecipitation assays were performed in J82 cells using a FOXC1 antibody (Cat# 8758, Cell Signaling Technology, Danvers, MA, USA) according to ENCODE standards, as previously described [27] using two biological replicates. ChIP-seq libraries were constructed using KAPA HyperPrep Kit (Roche) and NEXTflex DNA Barcodes (Bioo Scientific, Austin, TX, USA), and the sequencing was performed at the University of Southern California Molecular Genomics Core using NextSeq 550 platform for single-end sequencing, 75-bp read length, for about 50 million raw reads per sample. All ChIP-seq data were mapped to hg19 using bwa [25], and the peaks were called against input using MACS2 2.2.7.1 [26]. Reproducible peaks were called with the IDR tool, according to the ENCODE3 ChIP-seq pipeline (Encyclopedia of DNA Elements, https://www.encodeproject.org/chip-seq/ accessed on 20 October 2020) (Supplementary Table S2). FOXC1 and H3K27ac binding were visualized using deeptools [28].

### 2.6. Motif Analysis

Promoters are heavily enriched with conserved sequences (e.g., TATA box and CpG island consensus sequences), making it difficult to identify cell-type specific transcription factor putative binding sites. Therefore, to identify the motifs associated with transcription factors that are specifically enriched in SP or NSP cells, we performed a motif analysis in the enhancers. Enhancers were defined as the H3K27ac binding sites at least 2000 bp away from any transcription start sites (Gencode version 19), as previously done [20,29,30,31]. An accessible enhancer was defined as an enhancer with at least one accessible probe on the AcceSsslble assays. An SP-only enhancer was defined as an enhancer with at least one accessible probe in SP and zero accessible probes in NSP. The putative transcription factor binding sites on the enhancers were defined as the ENCODE DNase hypersensitivity sites on the enhancers within 500 bp from the accessible probes. The differential motif enrichment analysis was performed using HOMER v4.11 [32] via findMotifsGenome function, comparing the putative transcription factor binding sites in SP with the comparator (NSP) sequences as the background. De novo FOXC1 motif discovery was performed using HOMER findMotifsGenome function based on the top 500 FOXC1 ChIP-seq peaks on the enhancers using the default size of 200 bp near the peak summits.

### 2.7. FOXC1 Knockout

Knockout of FOXC1 was performed using CRISPR/Cas9 (lentiCRISPR v2, Cat# 52961, Addgene, Watertown, MA, USA). The lentiviral construct was courtesy of the Cui Lab at Cedars-Sinai Medical center [28]. The guide RNA (gRNA) sequence was 5′-GGGTGCGAGTACACGCTCAT-3′. The cells were then selected with puromycin 1–2 weeks before any experiments. Knockout clones were confirmed by Western blotting analysis.

### 2.8. Western Blotting

Proteins were extracted from human bladder cancer cells using a RIPA lysis buffer (Sigma-Aldrich), and the protein concentrations were determined by the BCA Protein Assay Kit (Bio-Rad, Hercules, CA, USA). Proteins were separated on 4–20% gradient gels and transferred to PVDF membranes using the iBlot Dry Blotting System (Thermo Fisher Scientific). Membranes were blocked in an Odyssey blocking buffer (LI-COR, Lincoln, NE, USA) and incubated with primary antibodies overnight at 4 °C. The primary antibodies were FOXC1 (1:1000, Cat# 8758, Cell Signaling Technology) and GAPDH (1:10,000, Cat# 2118, Cell Signaling Technology). The membranes were then incubated with goat anti-rabbit DyLight 800 secondary antibodies (1:10,000, Cat# 35571, Thermo Fisher Scientific) for 1 h at room temperature. Membranes were scanned using the Odyssey infrared imaging system (LI-COR).

### 2.9. Real-Time qPCR (RT-qPCR)

The total RNA was extracted from cells using the Direct-zol RNA Kit (Zymo Research) and was reverse-transcribed into single-stranded cDNAs using the qScript cDNA Synthesis Kit (Quanta BioSciences, Beverly, MA, USA). RT-qPCR was performed using Perfecta SYBR Supermix-IQ (Quanta BioSciences). FOXC1 primer sequences were forward 5′-CGGGTTGGAAAGGGATATTTA-3′ and reverse 5′-CAAAATGTTCTGCTCCTCTCG-3′. Quantitative PCR was performed in triplicate using the MyiQ single-color real-time PCR detection system (Bio-Rad) for 40 cycles at 95 °C for 10 s and 57 °C for 45 s. The iQ5 optical system software version 2.0 was used to analyze the results and was normalized by GAPDH and β-actin as the internal controls.

### 2.10. Flow Cytometry Analysis of FOXC1 after Cisplatin Treatment

J82 cells were seeded in triplicate in six-well plates the day before treatment. The next day, the cell culture medium was replaced with fresh medium containing cisplatin (Sigma-Aldrich) at 20µM versus equal volume PBS as the controls for 24 h of incubation. For flow cytometry, the cells were trypsinized, counted, and resuspended in prewarmed 10% FBS DMEM media at a concentration of 1 × 10^6^/mL. The cells were fixed with 10% formalin (Sigma-Aldrich) for 10 min at room temperature and permeabilized with PBS-0.1% Triton X-100 (Sigma-Aldrich) for 10 min below room temperature. After fixation and permeabilization, the cells were stained with FOXC1 antibody (1:100, Cat# PA5-18171, Thermo Fisher Scientific) and Alexa Fluor 546 secondary antibody (1:1000, Cat# A-11056, Thermo Fisher Scientific). Hoechst 33342 (Sigma-Aldrich) was used for nuclear staining. The samples were analyzed by FACSymphony (BD Biosciences, Franklin Lakes, NJ, USA).

### 2.11. Cell Viability Assays with Cisplatin Treatment

Here, 5000 cells were seeded in six duplicates in each well of 96-well plates the day before treatment. The next day, the cell culture medium was replaced with fresh medium containing cisplatin (Sigma-Aldrich) from 1000 µM to 0.5 µM using 1/3 serial dilutions. The surviving cell percentages were measured using an MTS assay (CellTiter 96 AQueous One Solution Cell Proliferation Assay, Promega, Madison, WI, USA) after 24 h using the PBS treated cells as the control. The concentrations of cisplatin leading to the biggest difference in cell survival between FOXC1 KO cell lines and controls were selected for presentation.

### 2.12. Statistical Analysis and Graphical Packages

All statistical analyses were performed in R versions 4.0.2, except the *p* values for motif enrichments, which were calculated by HOMER [32]. The *p* values and the statistical tests used to calculate them can be found throughout the text.

## 3. Results

### 3.1. Differential Accessibility at Regulatory Elements between SP and NSP Cells

**Figure 1:**
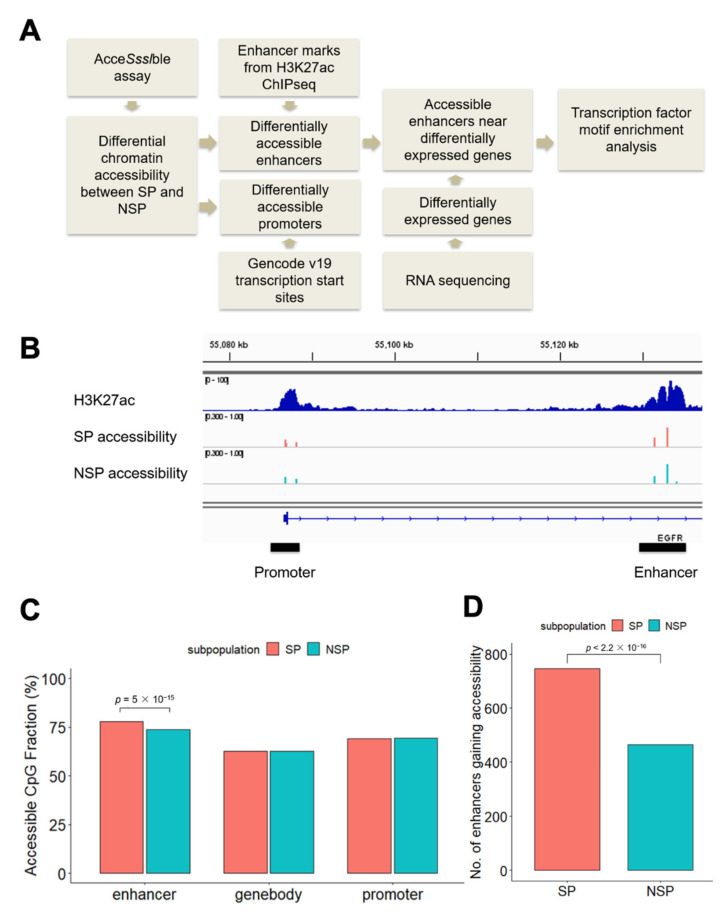
Differential accessibility at regulatory elements between SP and NSP cells. (**A**) Analysis workflow. (**B**) An intragenic enhancer within the EGFR gene from the set of the top 60,000 H3K27ac marks identified in J82 cells. (**C**) CpG sites at enhancers are more accessible in SP cells. The percentages of accessible CpG sites were calculated by dividing the number of accessible CpG sites (β-value increased > 0.3 after DNase treatment) by the total number of CpG sites in the given regions. The null hypothesis of the Chi-square test is that there is no relationship between the CpG sites accessibility (accessible versus not) and subpopulation (SP versus NSP). (**D**) SP cells have more enhancers that gain accessibility (defined as having at least one accessible CpG site compared with no accessible CpG sites in the same enhancer region in the counter subpopulation) than do NSP cells. The null hypothesis of the Chi-square test is that there is no relationship between the enhancer accessibility (gain versus not) and subpopulation (SP versus NSP).

To identify differentially accessible chromatin regions between drug-resistant SP and drug-sensitive NSP cells, we performed AcceSssIble assays in J82 to measure chromatin accessibility in the two cell subpopulations, as previously described [14,16]. The AcceSssIble assay utilizes CpG methyltransferase to convert unmethylated CpGs in open chromatin regions to methylated. Genome-wide open chromatin regions are identified by their increased methylation on CpGs after enzymatic treatment, using the Illumina Infinium Methylation EPIC BeadChip array platform. By combining the differential accessibility data with H3K27ac chromatin immunoprecipitation sequencing (ChIP-seq) and RNA sequencing (RNA-seq), we identified regulatory elements (promoters, enhancers) and target genes associated with the SP phenotype in J82 cells (Figure 1A). To examine chromatin accessibility at the promoters, we analyzed the CpG sites within a 2 kb window of transcription start sites. For enhancers, we examined regions located under H3K27ac marked sites outside of the promoters (>2 kb of transcription start sites) (Figure 1B). When we measured the genomic distribution of open chromatin regions, we found that enhancers were more accessible compared to promoters and gene bodies in J82 cells. Furthermore, more accessible enhancers were identified in SP cells, compared to NSP cells (Chi-square *p* = 5 × 10^−15^, Figure 1C, Supplementary Figure S1). Cells shifting to the SP phenotype gained greater enhancer accessibility across the genome than cells shifting to the NSP phenotype: Specifically, we identified 746 enhancers that are accessible in SP but not in NSP cells, significantly more than the 465 enhancers that are accessible in NSP but not in SP cells (Chi-square *p* < 2.2 × 10^−16^, Figure 1D). T24, another bladder cancer cell line, was used to validate our observation. We again observed that significantly more enhancers were gaining accessibility in SP cells than NSP cells (326 enhancers are accessible in SP but not in NSP cells, compared with 180 enhancers that are accessible in NSP but not in SP cells, Chi-square *p* < 2.2 × 10^−16^, Supplementary Figure S2). Our observation of greater differential accessibility at enhancers between SP and NSP cells motivated our subsequent investigation of transcription factors that drive the SP phenotype in bladder cancer by binding to enhancers.

### 3.2. Linking FOXC1 to Increased Enhancer Activity in SP Cells

**Figure 2:**
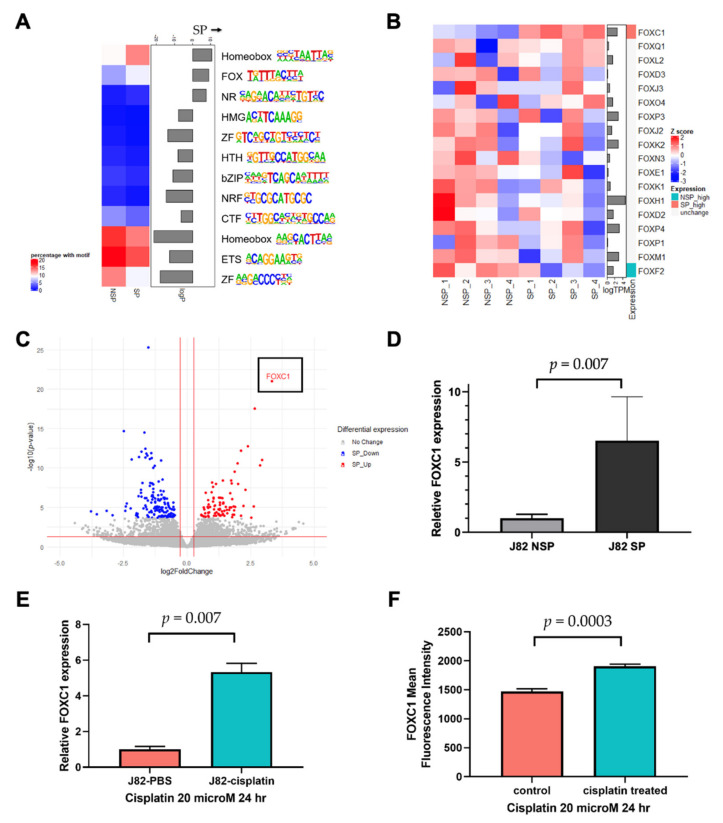
Linking FOXC1 to increased enhancer accessibility in drug-resistant bladder cancer cells. (**A**) The FOX family motif is enriched in accessible enhancers near overexpressed genes in SP cells. The color boxes on the left denote the percentage of DNase target region sequences, near accessible enhancer CpG sites, and differentially expressed genes, that contain the specific motifs. (**B**) RNA-seq identified FOXC1 as the only FOX family member overexpressed in SP cells; only genes with transcript per million (TPM) > 1 were considered expressed in our analysis. (**C**) The volcano plot showing FOXC1 is the most significantly overexpressed gene in SP cells. X-axis: log2 fold change comparing SP versus NSP. Gray: genes that are not statistically significantly changed; blue: underexpressed genes in SP cells; red: overexpressed genes in SP cells using *p* < 0.05 and fold change >30% cut-offs after filtering very low expressed genes. (**D**) qPCR confirmation of FOXC1 overexpression in SP cells. (**E**) RT-qPCR analysis demonstrated that FOXC1 mRNA increases after 24 h treatment of J82 cells with cisplatin. (**F**) The flow cytometry analysis demonstrated that FOXC1 protein expression increases after 24 h of treatment of J82 cells with cisplatin.

Our strategy to identify the key transcription factors driving the SP phenotype was to identify transcription factor motifs that are enriched at SP enhancers that are both accessible and associated with increased expression levels of their putative target genes. Because it cannot be known a priori which gene is regulated by a distal enhancer, we searched for enriched motifs on SP accessible enhancers located within one megabase of the genes overexpressed in SP cells (Supplementary Tables S1 and S3) using the HOMER motif database [32], and we found that the Homeobox (HOX), Forkhead box (FOX), and nuclear receptor (NR) family motifs were significantly enriched in SP cells (Figure 2A). Because members of a transcription factor family share a similar motif, we examined the RNA expression of all transcription factors belonging to the HOX, FOX, and NR families to identify the most likely family member that binds to the SP accessible enhancers. *FOXC1* and *NR4A3* were significantly overexpressed in SP cells (Supplementary Figure S3). FOXC1 is a transcription factor known to contribute to cancer progression [33,34]. Furthermore, the transcriptome analysis revealed that *FOXC1* was the only significantly overexpressed FOX family transcription factor in SP cells (Figure 2B). Notably, *FOXC1* was not only the most overexpressed transcription factor in SP cells, but also the single most overexpressed gene, compared to NSP cells, across the entire transcriptome, supporting the hypothesis that it may have an important function in SP cells (Figure 2C). Using RT-qPCR, we confirmed a significantly increased *FOXC1* expression in J82 SP cells, compared to NSP cells (*t*-test *p* = 0.007, Figure 2D). We also assessed the J82 *FOXC1* expression after cisplatin treatment. We found that the *FOXC1* mRNA expression was 5.3-fold higher after cisplatin treatment (*t*-test *p* = 0.002, Figure 2E). To test whether this increase was driven by the elimination of a small subset of FOXC1-low cells or the emergence of a small subset of FOXC1-high cells, we also conducted flow cytometry, which showed that cisplatin treatment increased the mean FOXC1 protein levels by 29.5% (*t*-test *p* = 0.0003, Figure 2F), and that it did so across the entire cell population (Supplementary Figure S4). These results indicate that bladder cancer cells exhibit an increased FOXC1 expression in drug resistant SP cells, and also in response to cisplatin exposure.

### 3.3. FOXC1 Binding Sites Are Significantly More Accessible in SP Cells

**Figure 3:**
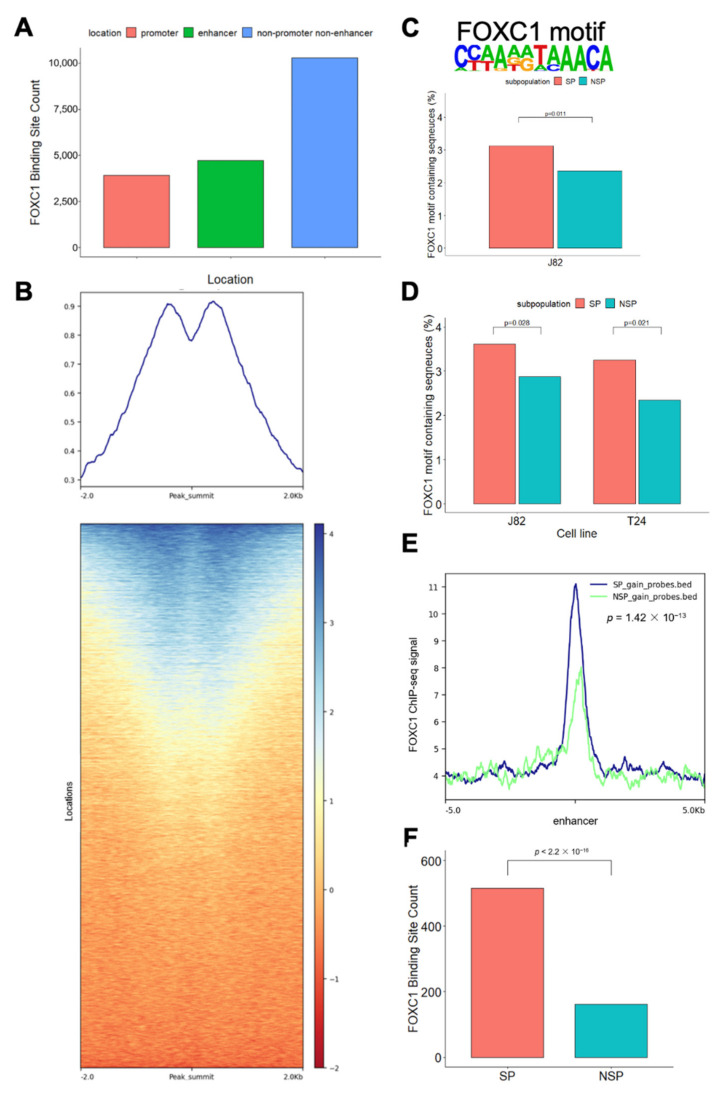
FOXC1 binding is associated with increased accessibility in SP cells. (**A**) Location of FOXC1 binding sites. Promoter FOXC1 binding sites are those within 2 kb of the transcription start sites. Enhancer FOXC1 binding sites are those under H3K27ac marked sites excluding the promoter sites. (**B**) Shown are the tag density plot and the heatmap of H3K27Ac ChIP-seq data centered on the genomic locations of the FOXC1 binding sites. (**C**) FOXC1 motif and its enrichment at accessible enhancers within one megabase of overexpressed genes in J82 SP cells. Percentages of the FOXC1 motif containing sequence were calculated by dividing the number of sequences containing the FOXC1 motif by the total number of sequences (sequences of the DNase target regions near accessible enhancer CpG sites and differentially expressed genes in one subpopulation) in the regions. (**D**) The FOXC1 motif is enriched at enhancers gaining accessibility in J82 and T24 SP cells. Percentages of FOXC1 motif containing sequence were calculated by dividing the sequences containing the FOXC1 motif by the total number of sequences (sequences of the DNase target regions on the enhancers near CpG sites gaining accessibility in one subpopulation) in the regions. (**E**) FOXC1 displays a higher binding to the enhancer CpG sites gaining accessibility in SP cells than to the enhancers gaining accessibility in NSP cells. Definition of CpG sites gaining accessibility: (1) the accessibility (β-value change after DNase treatment) is more than 0.3 (2) and the differences in accessibility between the subpopulations are more than 0.2. X-axis: distance to the enhancer CpG with increased accessibility. Y-axis: FOXC1 ChIP-seq signal. (**F**) Number of FOXC1 binding sites that gain accessibility in SP and NSP cells. FOXC1 binding sites that gain accessibility are defined as those with at least one accessible CpG site compared with no accessible CpG sites in the same FOXC1 binding sites in the counter subpopulation. The null hypothesis of the Chi-square test is that there is no relationship between the FOXC1 binding site accessibility (gain versus not) and subpopulation (SP versus NSP).

To characterize the function of FOXC1, we first performed FOXC1 ChIP-seq in J82 cells (Supplementary Figure S5). We observed FOXC1 binding at promoters and enhancers (H3K27ac ChIP-seq peaks that are located >2 kb from the transcription start site) as well as non-enhancer non-promoter regions (Figure 3A). We further investigated the H3K27ac mark near FOXC1 binding sites and noted a similar phenomenon, in that only half of the FOXC1 binding sites were marked by H3K27ac (Figure 3B). To further define the role of FOXC1 in the plasticity between the SP and NSP cells, we identified the enriched motif from FOXC1 ChIP-seq peaks and measured its enrichment levels in the SP versus NSP cells. We found that the FOXC1 motif is more enriched at accessible enhancers located within one megabase of genes overexpressed in SP cells (Figure 3C). To further investigate the role of FOXC1 across bladder cancer cell lines, we analyzed FOXC1 motif enrichment at enhancers gaining accessibility in T24 SP and NSP cells. Similar to J82 cells, the FOXC1 motif is significantly enriched at enhancers gaining accessibility in T24 SP cells (Figure 3D).

These motif-based findings were confirmed by the actual FOXC1 ChIP-seq data, which showed significantly more FOXC1 binding near enhancers gaining accessibility in SP cells (Kolmogorov–Smirnov test *p* = 1.42 × 10^−13^, Figure 3E). These enhancers, which gain accessibility in SP cells, are also more frequently located at the FOXC1 binding sites (Chi-square *p* = 0.037, Supplementary Figure S6). Across the genome, more FOXC1 binding sites gained accessibility in SP cells than FOXC1 binding sites gained accessibility in NSP cells (Chi-square *p* < 2.2 × 10^−16^, Figure 3F). Taken together, our observations support the association between FOXC1 binding and increased accessibility in SP cells.

### 3.4. FOXC1 Controls Genes Regulating Drug Resistance and Cancer Stemness

**Figure 4:**
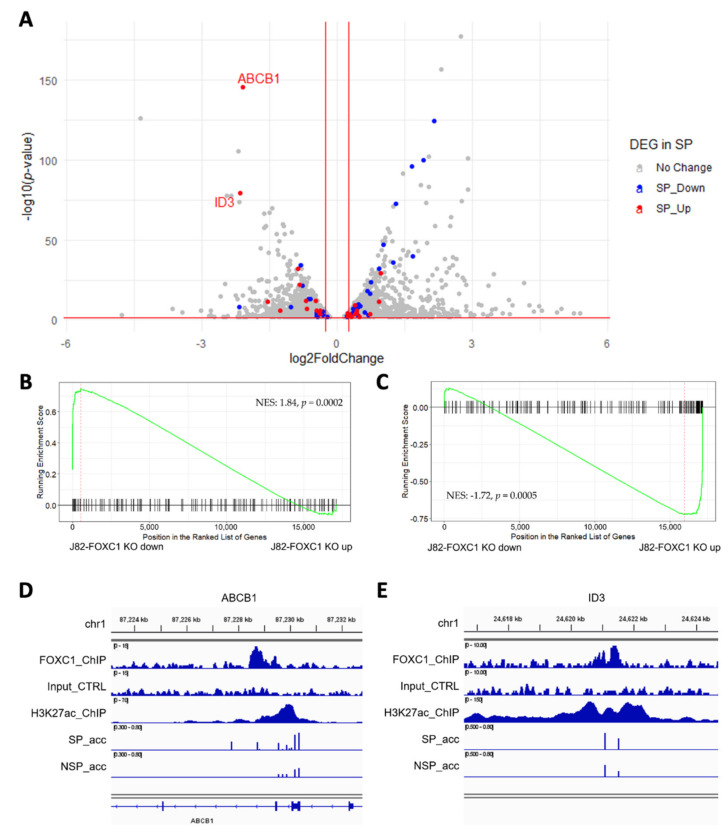
FOXC1 controls the genes regulating drug resistance and cancer stemness. (**A**) The volcano plot of differentially expressed genes upon FOXC1 knockout (FOXC1 KO) with the overlay of differentially expressed genes in SP and NSP (found from Figure 2C). X-axis: log2 fold change comparing FOXC1 KO versus control. The genes up-regulated in SP cells (red) are enriched among FOXC1 KO down-regulated genes (left), and the genes down-regulated in SP cells (blue) are enriched among FOXC1 KO up-regulated genes (right). (**B**) Geneset enrichment analysis (GSEA) shows genes down-regulated in SP cells are enriched among FOXC1 KO up-regulated genes. NES: normalized enrichment score. (**C**) GSEA shows genes upregulated in SP are enriched among FOXC1 KO down-regulated genes. (**D**) A FOXC1 binding site in the enhancer region located at the *ABCB1* gene body gains accessibility in SP cells. SP_acc: SP accessibility; NSP_acc: NSP accessibility. (**E**) A FOXC1 binding site in the enhancer region located at 770K upstream of the *ID3* gene gains accessibility in SP cells. SP_acc: SP accessibility; NSP_acc: NSP accessibility.

To investigate the FOXC1-regulated gene network, we performed RNA-seq on FOXC1 knockout J82 cells. We identified 1444 genes down-regulated and 1660 genes up-regulated by FOXC1 knockout (Figure 4A; gray dots, see also Supplementary Table S4). Across the transcriptome, we observed that genes up-regulated in SP are down-regulated by FOXC1 knockout (Figure 4A; blue dots). Gene set enrichment analysis also showed that up-regulated genes in SP cells are down-regulated in FOXC1 knockout cells (normalized enrichment score (NES): 1.84, *p* = 0.0002, Figure 4B). Taken together, these results suggest that FOXC1 regulates many of the genes that are overexpressed in SP cells. Conversely, down-regulated genes in SP cells were up-regulated by FOXC1 knockout (NES: −1.72, *p* = 0.0005, Figure 4C and red dots in Figure 4A). These observations suggest that transcriptional changes associated with the transition to the SP phenotype are orchestrated by the FOXC1-regulated program.

To identify candidate transcriptional targets of FOXC1 that contribute to the drug-resistant SP phenotype, we interrogated FOXC1 binding and accessibility changes at genes up-regulated in SP and down-regulated by FOXC1 knockout. *ABCB1*, a well-known ATP-binding cassette transporter associated with cisplatin resistance in bladder cancer [35,36], is the gene with the highest differential expression after FOXC1 knockout. There is a FOXC1 binding site under an H3K27ac marked site within the gene. In J82 cells, the CpG sites on and near the FOXC1 binding site showed increased accessibility in SP cells (Figure 4D).

Another gene, *ID3*, is down-regulated by FOXC1 knockout and up-regulated in SP cells. ID3 is an inhibitor of basic helix−loop−helix (bHLH) transcription factors and has essential roles in cancer stem cell renewal and drug resistance [37,38]. There is a FOXC1 binding site on an intergenic enhancer 770K upstream of the *ID3* transcription start site. While this enhancer is accessible overall in both SP and NSP cells, multiple CpG sites showed a higher level of accessibility in J82 SP cells (Figure 4E). Furthermore, multiple genes near this enhancer were down-regulated by FOXC1 knockout, including *PITHD1*, *FUCA1*, and *IFNLR1*, supporting the function of FOXC1 in regulating this enhancer.

To further strengthen our observation, we examined the accessibility change in these genomic areas using T24 cells. We found that the same areas near *ABCB1* and *ID3* also gain accessibility in T24 SP cells (Supplementary Figure S7). These observations suggested FOXC1 may regulate cisplatin resistance in bladder cancer via enhancer activation of multiple known drug-resistance genes such as *ABCB1* and *ID3*.

### 3.5. FOXC1 Regulates the Transition to the SP Phenotype and Cisplatin Resistance in Bladder Cancer Cells

**Figure 5:**
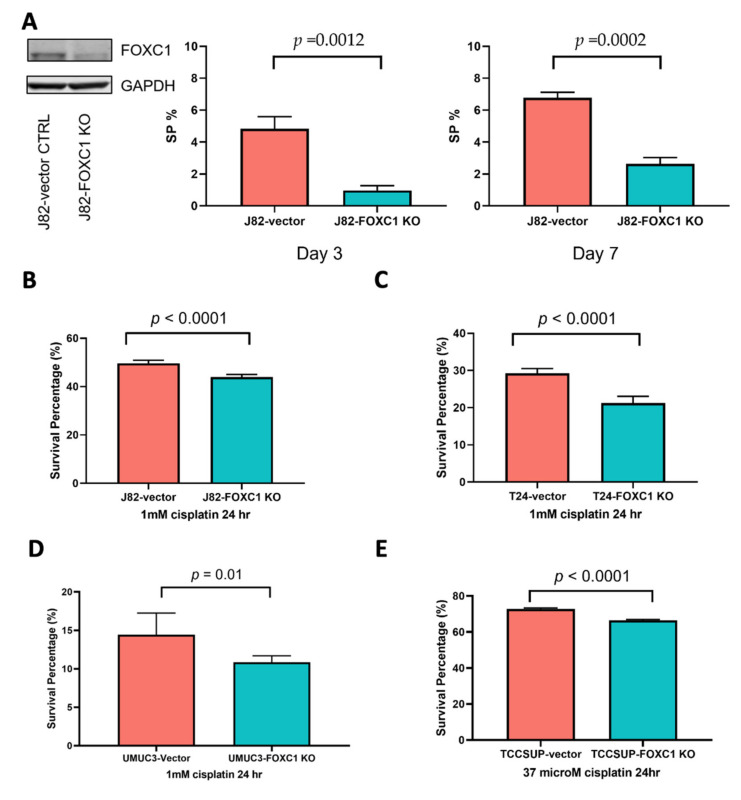
FOXC1 regulates the transition to the SP phenotype and cisplatin resistance in bladder cancer cells. (**A**) Left panel: Western blot showing a decrease in FOXC1 protein in the knockout cells; the percentage of SP cells is decreased at day 3 (middle panel) and day 7 (right panel) after FOXC1 knockout (FOXC1 KO) in J82 cells. FOXC1 KO decreases survival after cisplatin treatment of J82 (**B**), T24 (**C**), UMUC3 (**D**), and TCCSUP (**E**) cells. The uncropped blots of Figure 5A can be found in Supplementary Figure S11.

To further test the importance of FOXC1 in regulating bladder cancer cisplatin resistance, we performed a series of in vitro assays that investigated the function of FOXC1 in bladder cancer cells. We investigated the effect of FOXC1 knockout on the transition to the SP phenotype and drug resistance. In J82 cells, FOXC1 knockout significantly attenuates the emergence of the drug-resistant SP cells (*t*-test *p* = 0.0012 on day 3, and 0.0002 on day 7, Figure 5A, Supplementary Figure S8), without significantly impacting overall cell proliferation (Supplementary Figure S9). When cells were treated with cisplatin, J82 FOXC1 knockout cells had significantly less survival compared with the control cells (*t*-test *p* < 0.001, Figure 5B, Supplementary Figure S10A). Additional bladder cancer cell lines, including T24, UMUC-3, and TCCSUP, were tested to confirm the importance of FOXC1 in regulating cisplatin resistance. After FOXC1 knockout, all the cell lines demonstrated significantly decreased survival upon cisplatin treatment (Figure 5C–E, Supplementary Figure S10B–D). Although modest, the observed survival difference was consistent with the fact that FOXC1 knockout decreases drug-resistant SP cells, which comprise less than 10% of the overall cell population. In summary, FOXC1 potentiates transition to the drug-resistant phenotype across multiple bladder cancer cell lines.

## 4. Discussion

The canonical view of drug resistance focuses on genetic drivers, defined as the accumulation of DNA alterations conferring a selective growth advantage. However, there is rapidly accumulating evidence implicating epigenetic and other regulatory mechanisms in the development of resistance to various cancer therapeutic agents [39]. In this study, we aimed to characterize the epigenetic mechanisms driving the emergence of cisplatin resistance in bladder cancer. Using our previously described model of a cyclical transition between drug-resistant SP cells and drug-sensitive NSP cells in bladder cancer [4,5], we analyzed the chromatin accessibility of regulatory elements and found that enhancers had a higher level of differential accessibility than the promoters between SP and NSP cells. Using ChIP-seq and RNA-seq, we found that the transcription factor FOXC1 was not only highly overexpressed in SP relative to NSP cells, but also exhibited greater enhancer-binding in SP cells. FOXC1 knockout followed by RNA-seq revealed that FOXC1 positively regulates many of the same genes that are overexpressed in SP cells, some of which (e.g., *ABCB1* and *ID3*) have been shown to play a role in drug resistance and cancer stemness in previous reports [35,36,37,38]. Notably, these genes are located near FOXC1-bound enhancers that have increased accessibility in SP cells. In support of a functional importance of FOXC1 in bladder cancer, we found that treatment with cisplatin increased FOXC1 expression and that FOXC1 promoted conversion to the SP phenotype and cisplatin resistance in bladder cancer.

FOXC1 is a transcription factor known to play important roles in cancer progression. In lung cancer, FOXC1 expression is higher in tumors compared with normal tissues [40], and in breast cancer, FOXC1 expression is associated with a poor prognosis [41]. Moreover, an association between FOXC1 and chemotherapy resistance has been described in lung cancer [40,42], and FOXC1 has been shown to increase chemotherapy resistance and cancer stem cell properties in breast cancer [34]. FOXC1 downstream targets, such as MYC [43], LINC01123 [44], and MMP10 [45], have been reported to regulate tumor progression in various cancer types. In this study, we found that *ABCB1 and ID3* showed an increased expression in drug-resistant SP cells and became downregulated upon FOXC1 knockout. Furthermore, we identified FOXC1 binding at enhancers near these genes and these enhancers showed increased accessibility in SP cells. ABCB1 reduces intracellular concentrations of cytotoxic drugs, and is associated with chemotherapy resistance in bladder cancer [36] and with chemotherapy resistance and shorter survival in lung cancer [46]. ID3 belongs to the bHLH family, but lacks a DNA binding domain. It interacts with other bHLH proteins and prevents them from forming active heterodimers to bind DNA [47]. ID3 promotes the maintenance of cancer stemness and is associated with poor treatment outcomes in colon cancer and cholangiocarcinoma [37,48].

FOX family transcription factors bind a similar DNA sequence, but members of this large family have distinct expression patterns and regulate disparate biological processes [49]. One member of the FOX family, FOXA1, is a well-characterized pioneer transcription factor capable of priming the transition of chromatin from a condensed, inactive state to an accessible, transcriptionally competent one [50]. Given the conserved Forkhead domain amino acid sequence, the mechanism of FOXC1-induced enhancer activation may be similar to that of FOXA1 [51]. Indeed, the significant overlap between FOXC1 binding sites and increased chromatin accessibility in SP cells suggests that FOXC1 may act as a pioneer transcription factor in bladder cancer cells that transition to cisplatin resistance. Further supporting this possibility, we observed that half of the FOXC1 binding sites are outside promoter and enhancer regions, and are not marked by H3K27ac (Figure 3B,C). Furthermore, a recent report showed that *foxc1* is required for enhancer accessibility of genes controlling zebrafish facial cartilage development [52]. In our model, we speculate that when FOXC1 is overexpressed in SP cells or induced by cisplatin treatment, it binds to and transforms FOXC1-bound enhancers to their accessible and active state. This transition leads to the downstream activation of regulated target genes, including *ABCB1* and *ID3*, driving drug resistance in bladder cancer.

To the best of our knowledge, this is the first study describing a role for FOXC1 in regulating bladder cancer resistance to cisplatin. Our data suggest that the therapeutic effects of chemotherapy may be augmented by co-targeting the FOXC1-regulated network. Although there are no direct FOXC1 inhibitors described to date, previous reports have indicated that FOXC1 is regulated by the phosphatidylinositol 3-kinase (PI3K)-AKT signaling [53], a pathway that is being aggressively targeted with multiple therapeutics across cancer types. Consistent with this possible linkage between FOXC1, the AKT pathway, and cisplatin resistance of SP cells, we have previously shown that an AKT inhibitor can reduce the transition to the SP phenotype and decrease cisplatin resistance in bladder cancer [5]. Therefore, we suggest that therapeutic agents targeting the PI3K-AKT pathway could be used concomitantly with chemotherapy to inhibit FOXC1 signaling and suppress the emergence of drug resistance.

## 5. Conclusions

Spontaneous transition to a more drug-resistant phenotype is a mutation-independent mechanism of cisplatin resistance in bladder cancer. Using H3K27ac ChIP-seq and AcceSssIble assay, we discovered that bladder cancer cells with a drug-resistant phenotype have more accessible enhancers. The enhancers gaining accessibility in the drug-resistant phenotype are differentially bound by FOXC1, a transcription factor significantly overexpressed in the drug-resistant phenotype. Consistent with a role in regulating drug resistance, FOXC1 expression is increased upon cisplatin treatment, whereas FOXC1 knockout attenuates the emergence of the resistance phenotype and reduces cisplatin resistance in vitro. Collectively, these findings suggest that FOXC1 regulates phenotypic plasticity by promoting a mutation-independent shift towards cisplatin resistance in bladder cancer. As such, targeting the FOXC1-regulated transcriptional network concurrently with cisplatin therapy may constitute a novel therapeutic avenue that merits further investigation.

## Data Availability

The array-based DNA methylation data were previously published and deposited in the GEO repository under the accession number GSE123610. RNA-seq and ChIP-seq data are available at GEO under the accession number GSE169336. Additional data supporting our findings are available from the corresponding authors upon reasonable request.

## Supplementary Materials

The following supporting information can be downloaded at: https://www.mdpi.com/article/10.3390/cancers14071717/s1. Supplementary Figure S1: Accessibility difference between SP and NSP is greater at enhancers than at promoters. Figure S2: SP cells have more enhancers that gain accessibility than do NSP cells in T24. Figure S3: *FOXC1* and *NR4A3* are significantly overexpressed in SP cells. Figure S4: FOXC1 binding sites are identified genome-wide in J82 cells. Figure S5: Significantly more enhancer CpG sites gaining accessibility in SP are bound by FOXC1. Figure S6: T24 SP cells also gain accessibility in the enhancer CpG sites near *ABCB1* and *ID3*. Figure S7: Representative flow cytometry plots showing an overall shift toward higher FOXC1 protein expression after cisplatin treatment of J82 cells. Figure S8: Representative flow cytometry plots showing that FOXC1 knockout decreased SP population in J82 cells. Figure S9: FOXC1 knockout did not significantly change proliferation. Figure S10: FOXC1 knockout decreased cisplatin resistance across multiple bladder cancer cell lines. Figure S11: Uncropped western blot for FOXC1 protein expression in J82 vector control and J82 FOXC1 KO cells. Figure S12: Uncropped western blot for FOXC1 protein expression in T24 vector control and T24 FOXC1 KO cells. Figure S13: Uncropped western blot for FOXC1 protein expression in TCCSUP vector control, TCCSUP FOXC1 KO, UMUC3 vector control, and UMUC3 FOXC1 KO cells. Supplementary Table S1: J82 H3K27ac ChIP-seq peaks. Table S2: J82 FOXC1 ChIP-seq peaks. Table S3: SP NSP differentially expressed genes. Table S4: FOXC1 Knockout differentially expressed genes. Table S5: Survival percentages for the cisplatin dose-response curves in J82 and J82 FOXC1 KO cells. Table S6: Survival percentages for the cisplatin dose-response curves in T24 and T24 FOXC1 KO cells. Table S7: Survival percentages for the cisplatin dose-response curves in UMUC3 and UMUC3 FOXC1 KO cells. Table S8: Survival percentages for the cisplatin dose-response curves in TCCSUP and TCCSUP FOXC1 KO cells.
