# Supplementary material for: FOXC1 Binds Enhancers and Promotes Cisplatin Resistance in Bladder Cancer
Source: Cancers (Basel). 2022 Mar 28;14(7):1717. doi: 10.3390/cancers14071717 (PMC8996937; doi:10.3390/cancers14071717)
Supplement: Supplementary file 1 [file cancers-14-01717-s001.zip › Supplementary_figures_cancers.pdf]

FOXC1 binds enhancers and promotes cisplatin resistance in bladder cancer

Yi-Tsung Lu, Tong Xu, Maheen Iqbal, Tien-Chan Hsieh, Zhifei Luo, Gangning Liang, Peggy J. Farnham, Suhn K. Rhie and Amir Goldkorn

Supplementary Figures

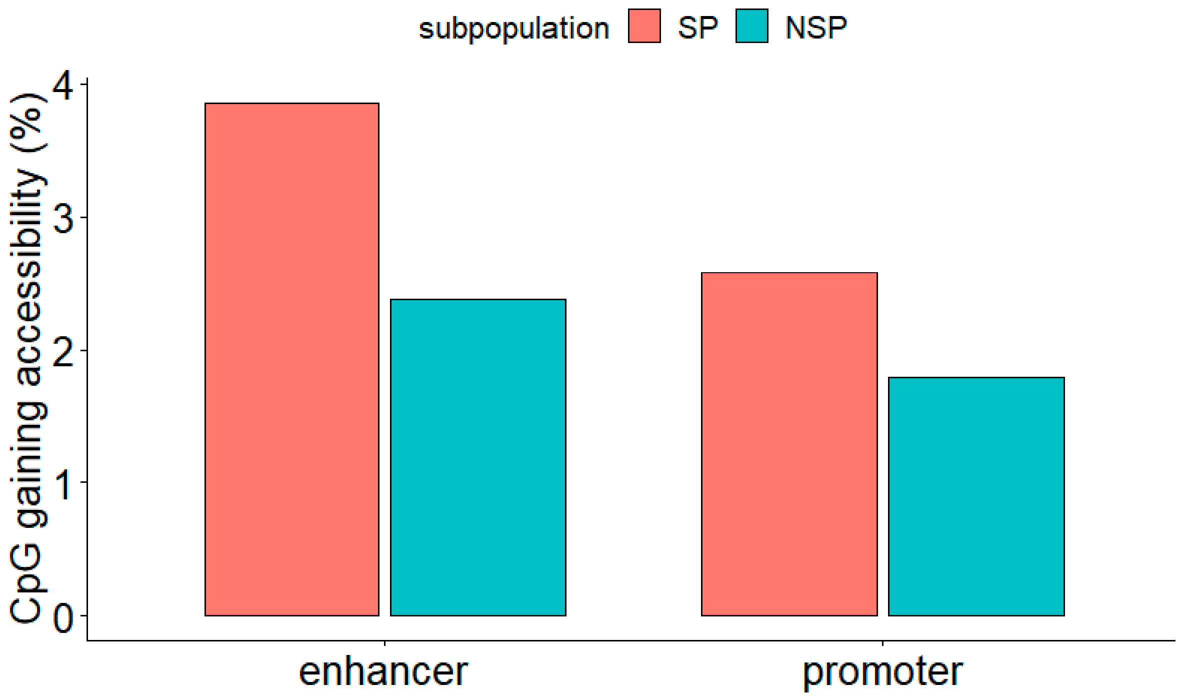

Figure S1. Accessibility difference between SP and NSP is greater at enhancers than at promoters. CpG sites gaining accessibility were defined as CpG sites becoming accessible (β-value increased > 0.3 after M.SssI treatment) in one subpopulation but not the other.

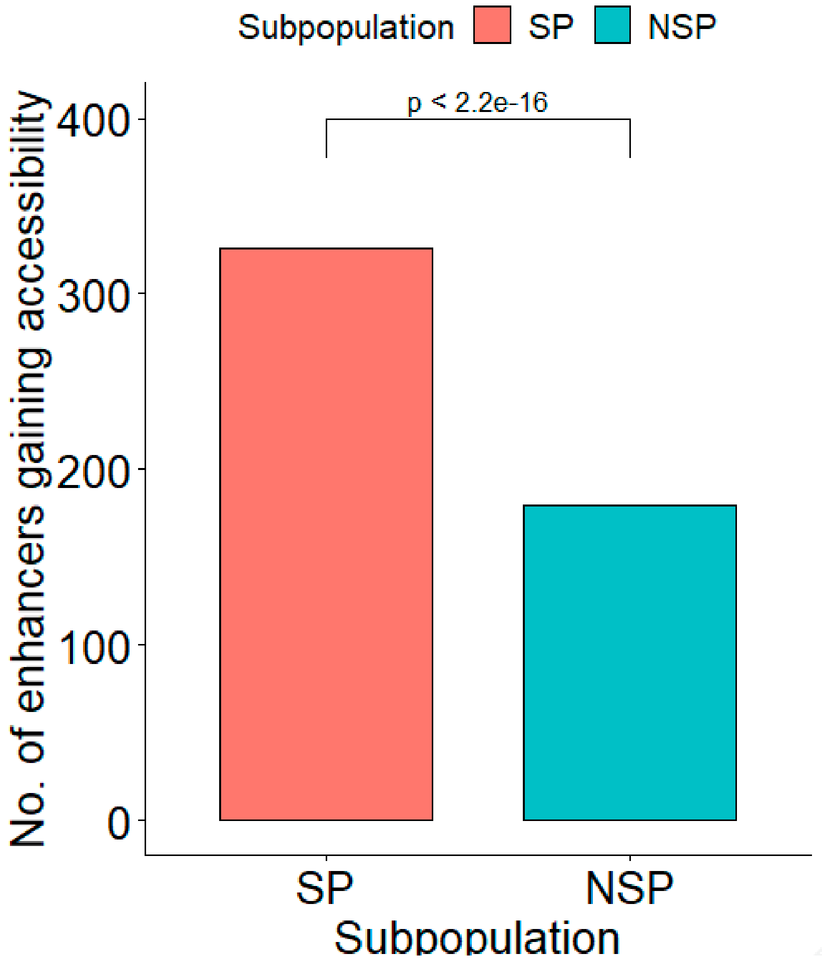

Figure S2. SP cells have more enhancers that gain accessibility than do NSP cells in T24. The gain of accessibility is defined as having at least one accessible CpG site as compared with no accessible CpG sites in the same enhancer region in the counter sub-population. The null hypothesis of the chi-square test is that there is no relationship between the enhancer accessibility (gain versus not) and subpopulation (SP versus NSP).

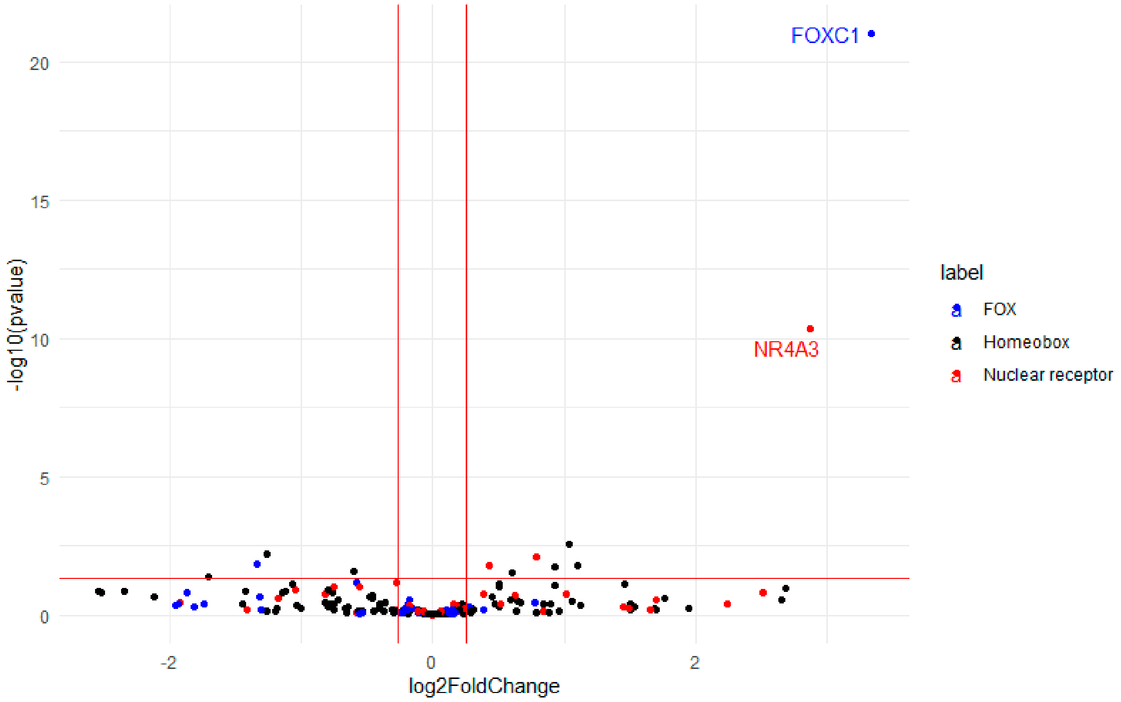

Figure S3. FOXC1 and NR4A3 are significantly overexpressed in SP cells. Volcano plot of differential expression levels between SP and NSP for all transcription factors in the homeobox, FOX, and nuclear factor families between SP and NSP cells.

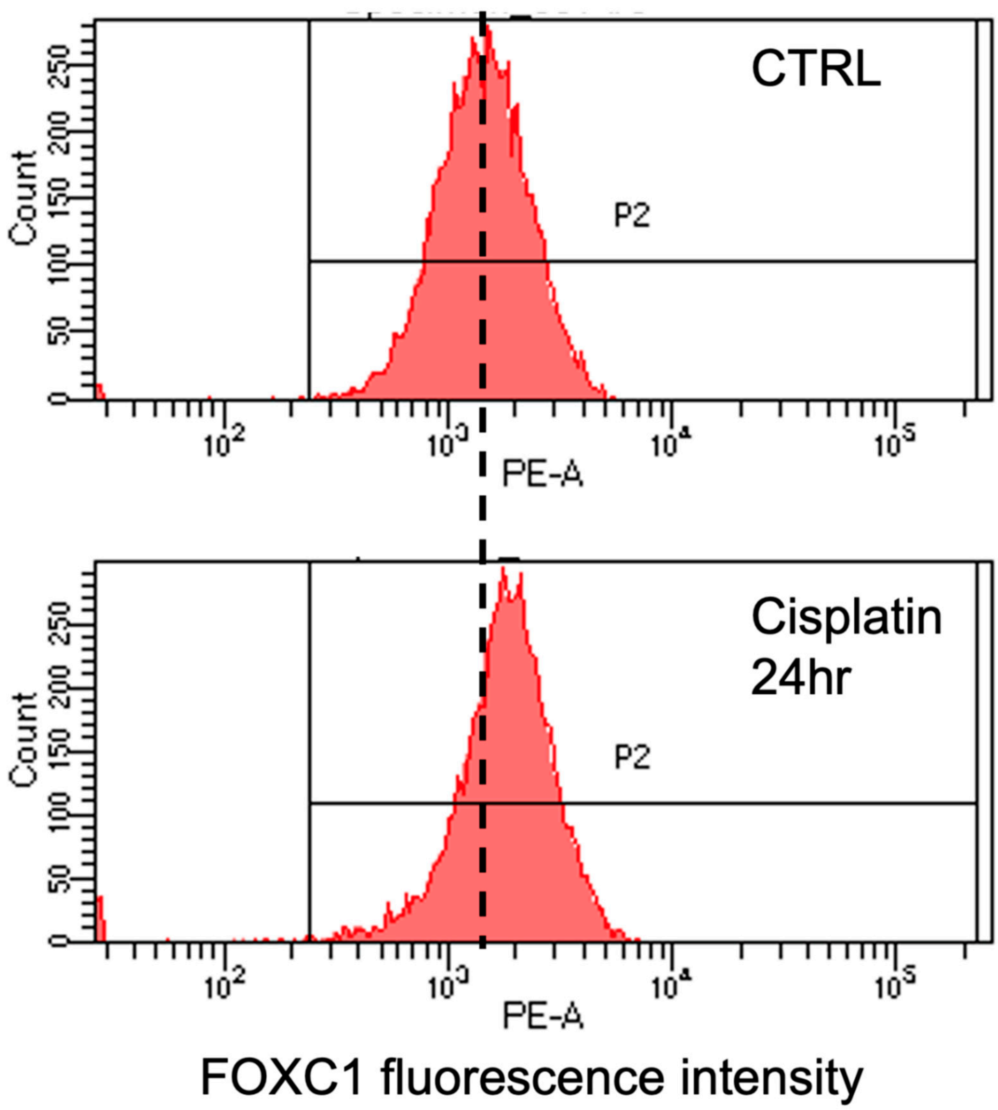

Figure S4. Representative flow cytometry plots showing an overall shift toward higher FOXC1 protein expression after cisplatin treatment of J82 cells.

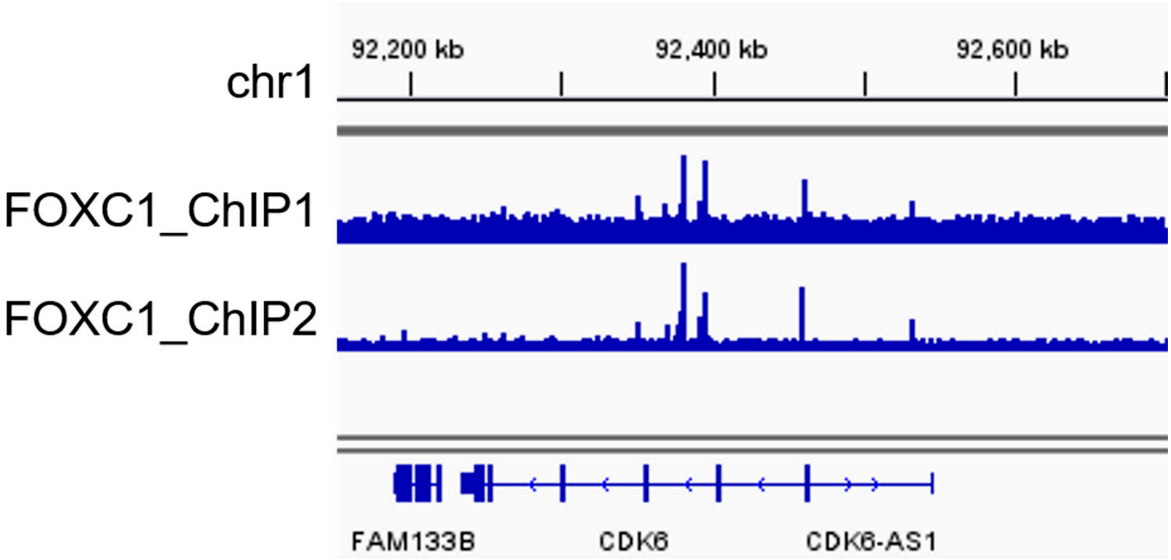

Figure S5. FOXC1 binding sites are identified genome-wide in J82 cells. An example FOXC1 binding site near the *CDK6* gene, demonstrating FOXC1 ChIP-seq peak consistency between two replicates.

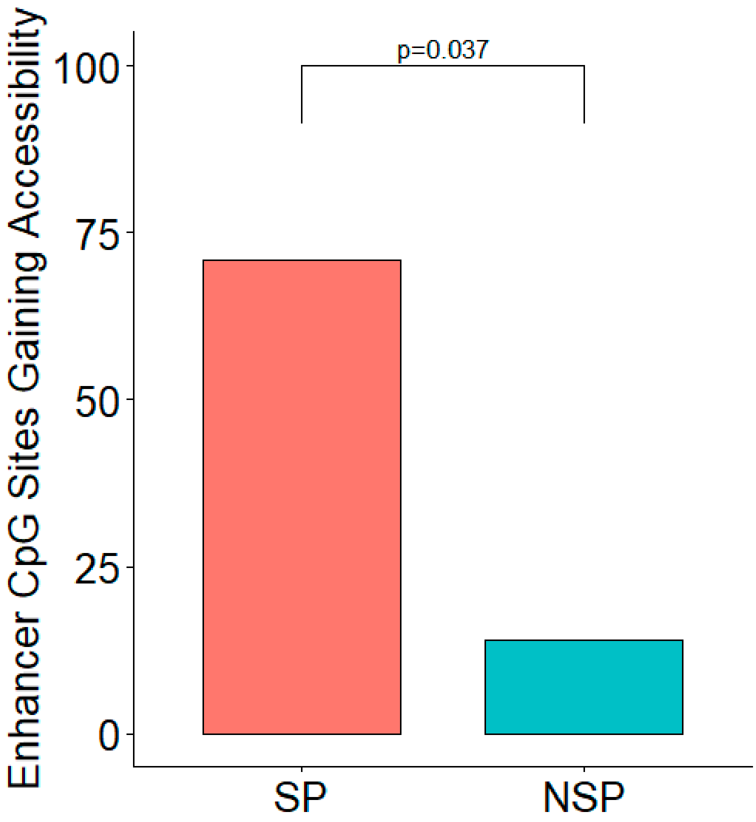

Figure S6. Significantly more enhancer CpG sites gaining accessibility in SP are bound by FOXC1. Definition of CpG sites gaining accessibility: 1. The accessibility (β-value change after M.SssI treatment) is more than 0.3; 2. The differences in accessibility between the subpopulations are more than 0.2. The Y-axis: the number of enhancer CpG sites with increased accessibility. The null hypothesis of the chi-square test is that there is no relationship between the FOXC1 binding sites and subpopulations among the enhancer CpG sites gaining accessibility.

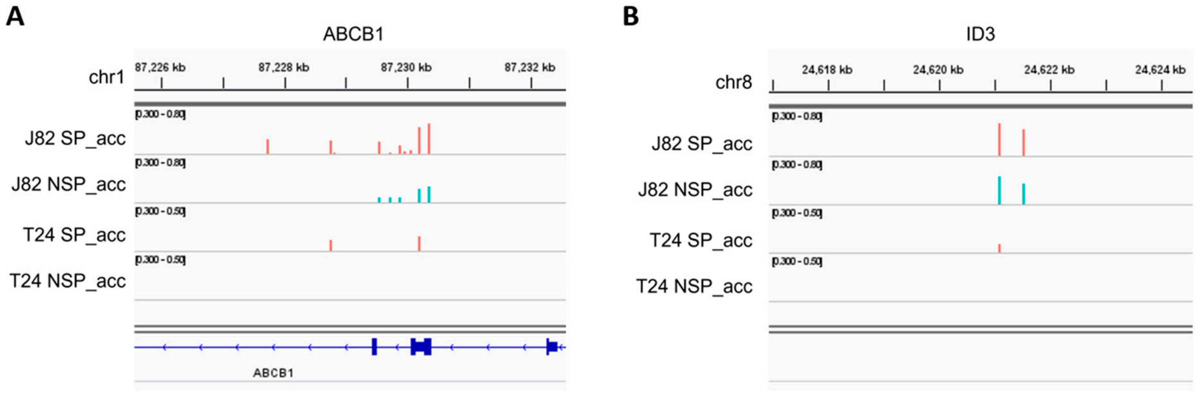

Figure S7. T24 SP cells also gain accessibility in the enhancer CpG sites near *ABCB1* and *ID3*. (A) The enhancer located within the *ABCB1* gene gained accessibility in both J82 and T24 SP cells. (B) The enhancer near the *ID3* gene also gained accessibility in both J82 and T24 SP cells.

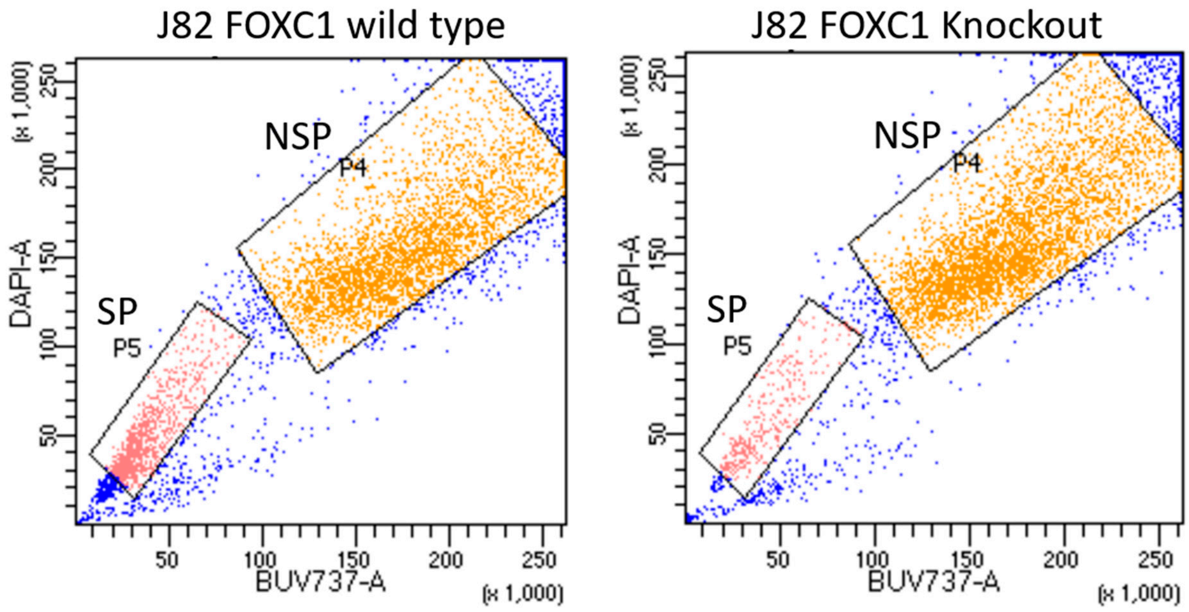

Figure S8. Representative flow cytometry plots showing that FOXC1 knockout decreased SP population in J82 cells.

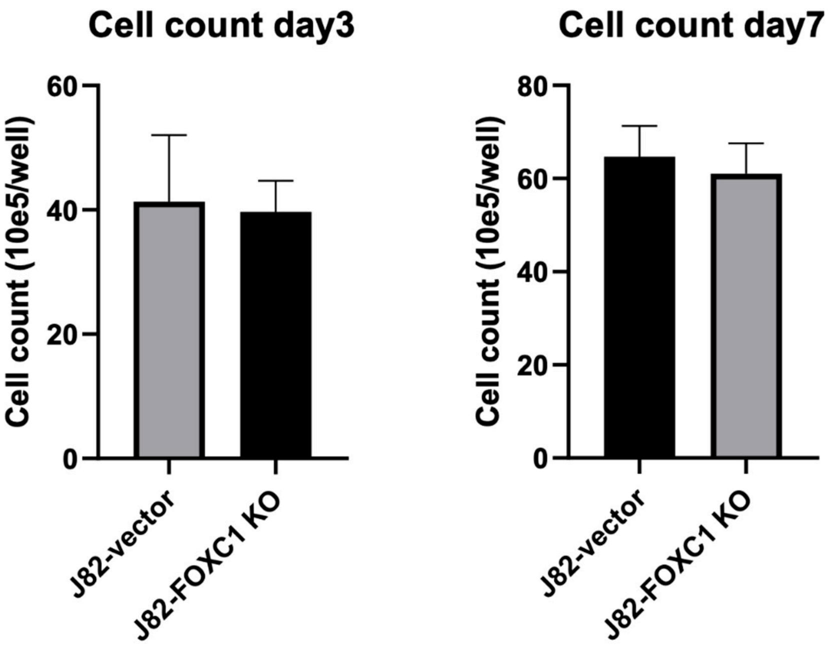

Figure S9. FOXC1 knockout did not significantly change proliferation. Cell counts at the time of the SP assays were not significantly different between J82-FOXC1 knockout (n=3) and J82-vector control (n=3).

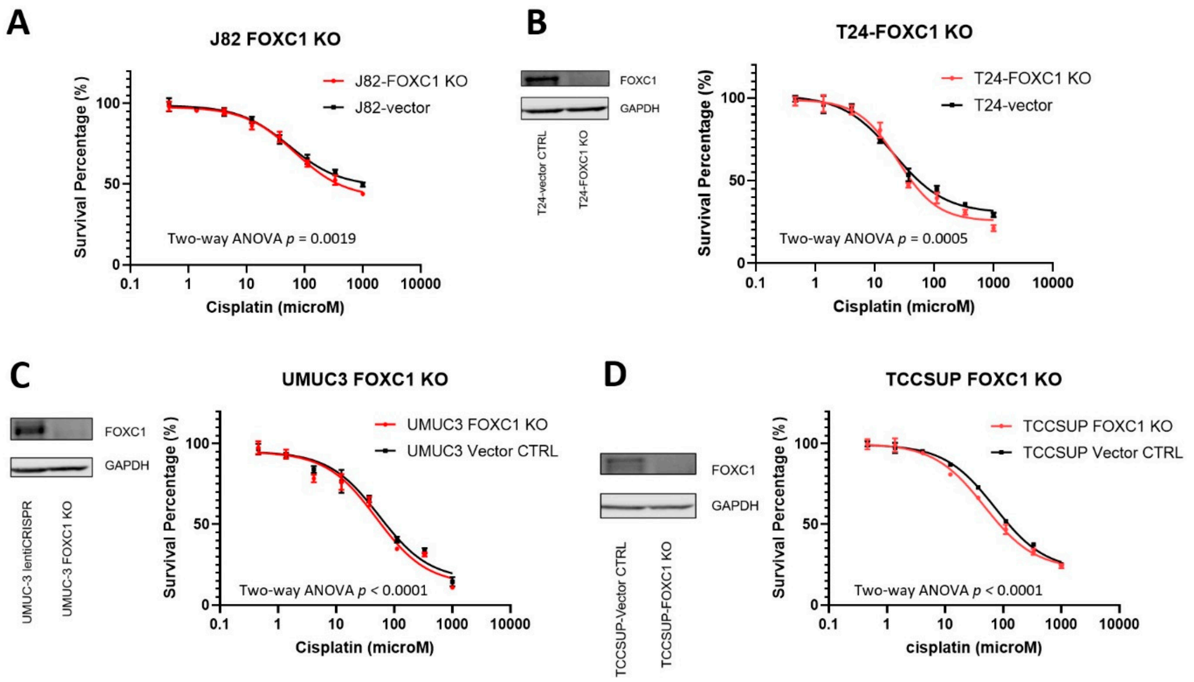

Figure S10. FOXC1 knockout decreased cisplatin resistance across multiple bladder cancer cell lines. Dose-response curves for cisplatin treatment after FOXC1 knock-out in (**A**) J82 cells, (**B**) T24 cells, (**C**) UMUC3 cells, and (**D**) TCCSUP cells (n=6 for all the experiments). Two-way ANOVA is used to compare the means of the survival rates in both cell lines. Survival percentage for each cisplatin concentration and each cell line can be found in Supplementary Tables S5-8. The uncropped blots of Supplementary Figure 10B-D can be found in Supplementary Figure S12-13.

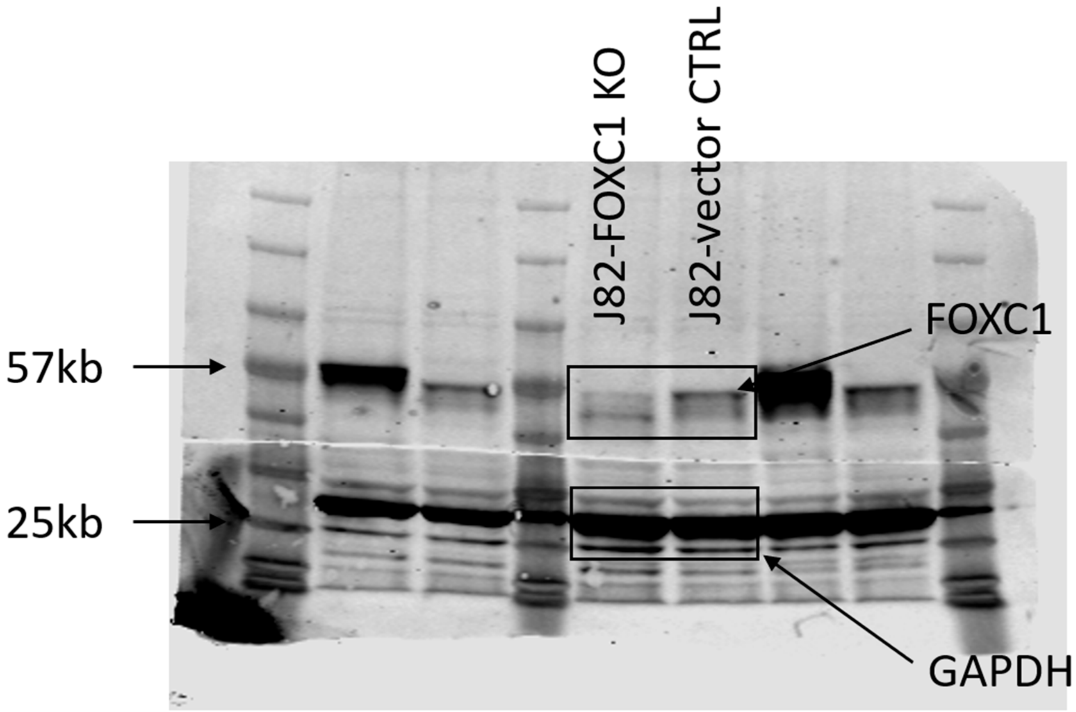

Figure S11. Uncropped western blot for FOXC1 protein expression in J82 vector control and J82 FOXC1 KO cells.

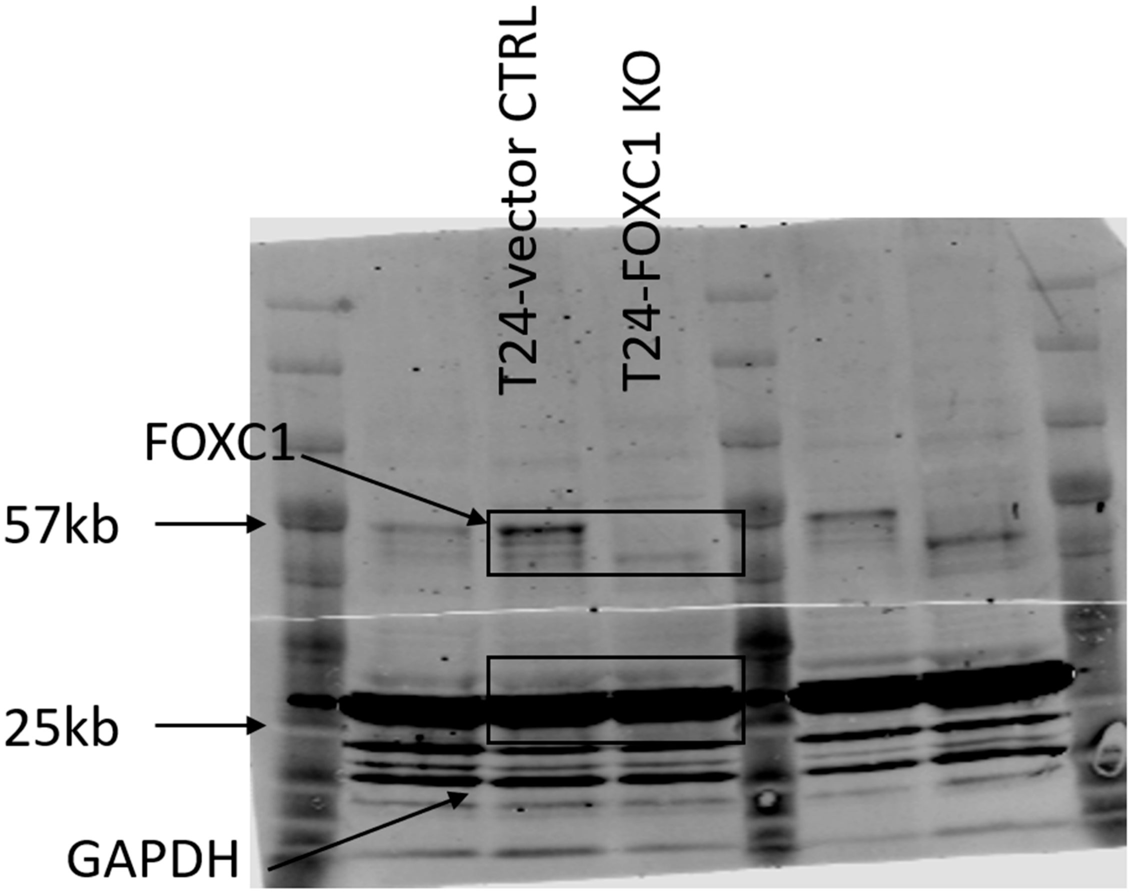

Figure S12. Uncropped western blot for FOXC1 protein expression in T24 vector control and T24 FOXC1 KO cells.

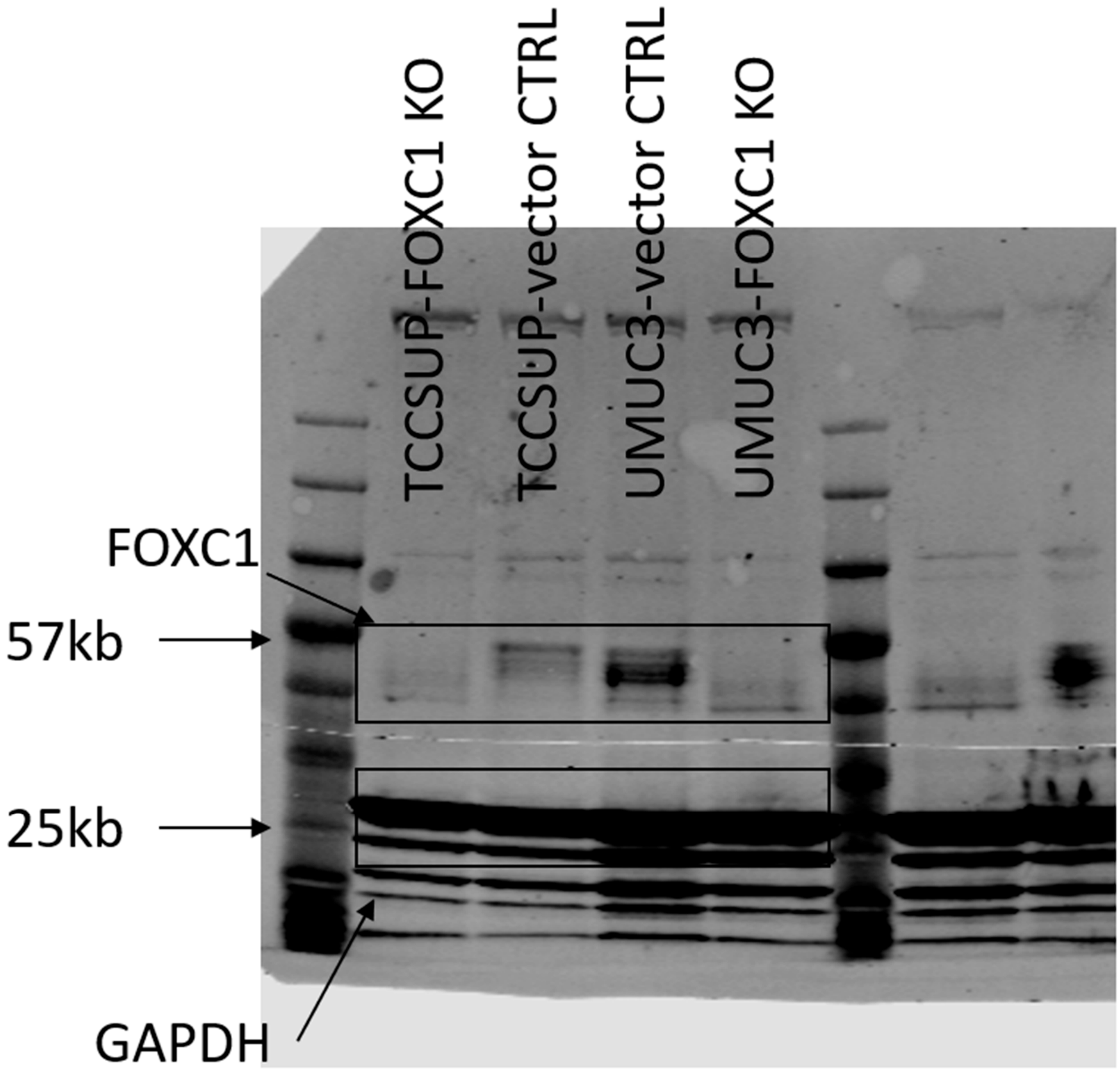

Figure S13. Uncropped western blot for FOXC1 protein expression in TCCSUP vector control, TCCSUP FOXC1 KO, UMUC3 vector control, and UMUC3 FOXC1 KO cells.
